# Densely Populated Water Droplets in Heavy-Oil Seeps

**DOI:** 10.1128/AEM.00164-20

**Published:** 2020-05-19

**Authors:** M. Pannekens, L. Voskuhl, A. Meier, H. Müller, S. Haque, J. Frösler, V. S. Brauer, R. U. Meckenstock

**Affiliations:** a Environmental Microbiology and Biotechnology, University of Duisburg-Essen, Essen, Germany; b Department of Physics, Faculty of Science and Technology, The University of The West Indies, St. Augustine, Trinidad and Tobago; Chinese Academy of Sciences

**Keywords:** 16S rRNA sequencing, active degradation, bitumen, core community, life in oil, microhabitat, oil degradation, oil reservoir

## Abstract

Most of the microbial degradation in oil reservoirs is believed to take place at the oil-water transition zone (OWTZ). However, a recent study indicates that there is microbial life enclosed in microliter-sized water droplets dispersed in heavy oil of Pitch Lake in Trinidad and Tobago. This life in oil suggests that microbial degradation of oil also takes place in water pockets in the oil-bearing rock of an oil leg independent of the OWTZ. However, it is unknown whether microbial life in water droplets dispersed in oil is a generic property of oil reservoirs rather than an exotic exception. Hence, we took samples from three heavy-oil seeps, Pitch Lake (Trinidad and Tobago), the La Brea Tar Pits (California, USA), and an oil seep on the McKittrick oil field (California, USA). All three tested oil seeps contained dispersed water droplets. Larger droplets between 1 and 10 μl revealed high cell densities of up to 10^9^ cells ml^−1^. Testing for ATP content and LIVE/DEAD staining showed that these populations consist of active and viable microbial cells with an average of 60% membrane-intact cells and ATP concentrations comparable to those of other subsurface ecosystems. Microbial community analyses based on 16S rRNA gene amplicon sequencing revealed the presence of known anaerobic oil-degrading microorganisms. Surprisingly, the community analyses showed similarities between all three oil seeps, revealing common OTUs, although the sampling sites were thousands of kilometers apart. Our results indicate that small water inclusions are densely populated microhabitats in heavy oil and possibly a generic trait of degraded-oil reservoirs.

**IMPORTANCE** Our results confirmed that small water droplets in oil are densely populated microhabitats containing active microbial communities. Since these microhabitats occurred in three tested oil seeps which are located thousands of kilometers away from each other, such populated water droplets might be a generic trait of biodegraded oil reservoirs and might be involved in the overall oil degradation process. Microbial degradation might thus also take place in water pockets in the oil-bearing oil legs of the reservoir rock rather than only at the oil-water transition zone.

## Abstract (precis)

Our results confirmed that small water droplets in oil are densely populated microhabitats containing active microbial communities. Since these microhabitats occurred in three tested oil seeps which are located thousands of kilometers away from each other, such populated water droplets might be a generic trait of biodegraded oil reservoirs and might be involved in the overall oil degradation process. Microbial degradation might thus also take place in water pockets in the oil-bearing oil legs of the reservoir rock rather than only at the oil-water transition zone.

## INTRODUCTION

The world’s oil reservoirs are dominated by heavy oil (hereinafter referred to as oil) and bitumen, since anaerobic microorganisms have degraded the oil in the absence of molecular oxygen over geological time scales (1, 2). However, the metabolic processes and rates of biodegradation in deep oil reservoirs remain vague due to a lack of sufficient samples and the long geological timescales in which the degradation takes place (3). Several studies have shown that microbial abundance and biological degradation rates are highest at the so-called oil-water transition zone (OWTZ), i.e., the oil-water interface between an oil leg, the oil-bearing layer of an oil reservoir, and the underlying water leg (1). With increasing distance from this transition zone, biodegradation should be limited by lack of water, electron acceptors, and dissolved inorganic nutrients like sulfate, phosphorus, and nitrogen compounds. Hence, it is commonly assumed that no degradation takes place within the oil leg itself (1, 4). However, indicators for microbial life are found in almost all oil and water samples from reservoirs and even in heavy-oil or asphalt seeps with temperatures up to 82°C (5–14). This includes the largest natural asphalt lake, Pitch Lake, located on the island of Trinidad, Trinidad and Tobago. In this natural oil seep, Meckenstock et al. discovered complex microbial communities inhabiting tiny water droplets, 1 to 3 μl in volume, suspended in the oil phase, hereinafter termed water droplets or droplets (15). Since geochemical and isotopic analysis of the droplet water revealed a deep subsurface origin, it was concluded that the water droplets, containing indigenous microbiota, ascended directly from the oil reservoir. In fact, water-wet oil reservoirs contain water either as thin water films covering the sand grains and rock matrix or in water-filled pockets (11). Analysis of the 16S rRNA genes from single water droplets identified, among others, typical oil-degrading bacteria like *Bacteroidales*, *Rhodospirillales*, and *Sphingomonadales*, as well as methanogenic archaea, indicating hydrogenotrophic methanogenesis as the terminal electron-accepting process (15, 16). The microbial activity in the water droplets indicated that biodegradation in oil reservoirs is not restricted only to the oil-water transition zone. Furthermore, the biodegradation might take place directly within the oil leg, resulting in an increasing oil-water interface and potentially greater overall oil degradation. These water droplets provide a unique opportunity to get insights into the microbial life and degradation processes in the deep subsurface of oil reservoirs.

Nevertheless, it remained unclear whether such microbial communities entrapped in water droplets are a generic feature of oil reservoirs or only a single observation from Pitch Lake in Trinidad and Tobago. Hence, we sampled two additional natural oil seeps and studied the microbial composition of single water droplets. Furthermore, we elucidated basic features of these microbial communities, including the cell density, live/dead rates of single cells, metabolic activity, whether the microbes were living planktonically in the droplet lumen or arranged in biofilms at the oil-water interface of each droplet, and finally, the microbial community composition as a tool to identify typical oil-degrading microorganisms.

## RESULTS

### Distribution and density of microorganisms in water droplets.

**FIG 1:**
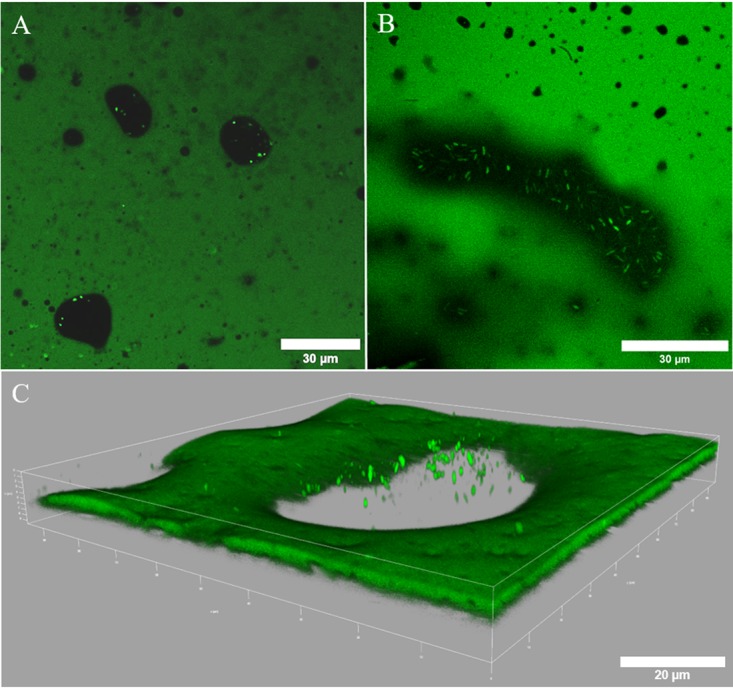
CLSM fluorescence images of natural water droplets (black) dispersed in oil (green) from McKittrick (A, C) and La Brea (B) oil samples. Bright green dots represent microbial cells stained with Syto 9. (A, B) Two-dimensional view of different water droplets. (C) Three-dimensional view of different water droplets.

**FIG 2:**
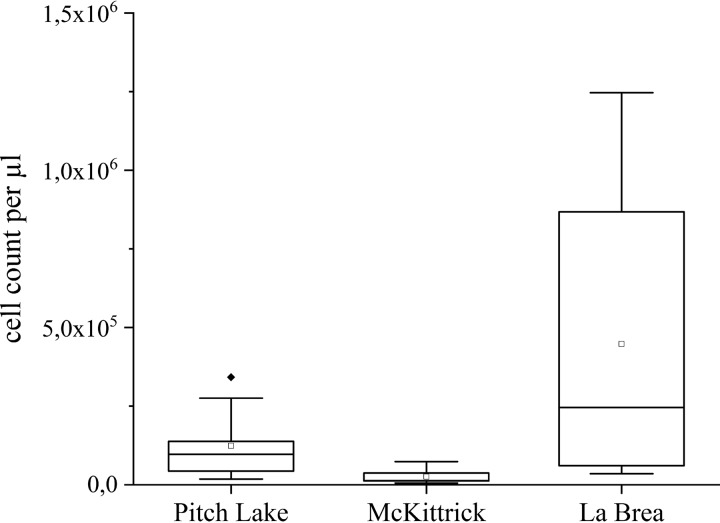
Box plots of total cell counts of isolated water droplets from the three different oil seeps: Pitch Lake (Trinidad and Tobago), McKittrick (CA, USA), and La Brea Tar Pits (CA, USA). In total, 30 droplets (10 of each oil seep) were counted with a Thoma chamber.

In order to determine the localization of microorganisms in water droplets enclosed in oil from Pitch Lake in Trinidad, we performed confocal laser scanning microscopy (CLSM), which revealed small water inclusions dispersed in the oil (Fig. 1). Pictures of Syto 9-stained specimens clearly showed microorganisms in these droplets, but cells were only found in water inclusions larger than 10 to 20 μm in diameter (Fig. 1). Due to the addition of the staining solution, the actual droplet volume of the droplets was artificially enhanced. Total cell counts of the lumen of isolated water droplets revealed that most droplets contained microbial cells, with abundances ranging from 5.6 × 10^3^ to 1.2 × 10^6^ cells μl^−1^ (Fig. 2). The average cell numbers ranged from 2.6 × 10^4^ cells μl^−1^ (*n* = 10) in the McKittrick water droplets, over 1.2 × 10^5^ cells μl^−1^ (*n* = 10) in the ones from Pitch Lake, to 4.5 × 10^5^ cells μl^−1^ (*n* = 10) in La Brea Tar Pit droplets. The highest cell density in a single droplet was found in La Brea oil, with 1.2 × 10^6^ cells μl^−1^. According to the fluorescence microscopy results mentioned above, it is likely that some cells were attached at the oil-water interface and were not detected in this counting. The observed cells differed in size, shape, and composition, indicating diverse communities inside different droplets. The most abundant morphologies were rods and diplobacilli, respectively, but cocci, diplococci, and filamentous microorganisms were also observed. The epifluorescence counting of filtered droplet water (data not shown) confirmed the counting via Thoma chamber.

### Water droplets contain living cells.

**FIG 3:**
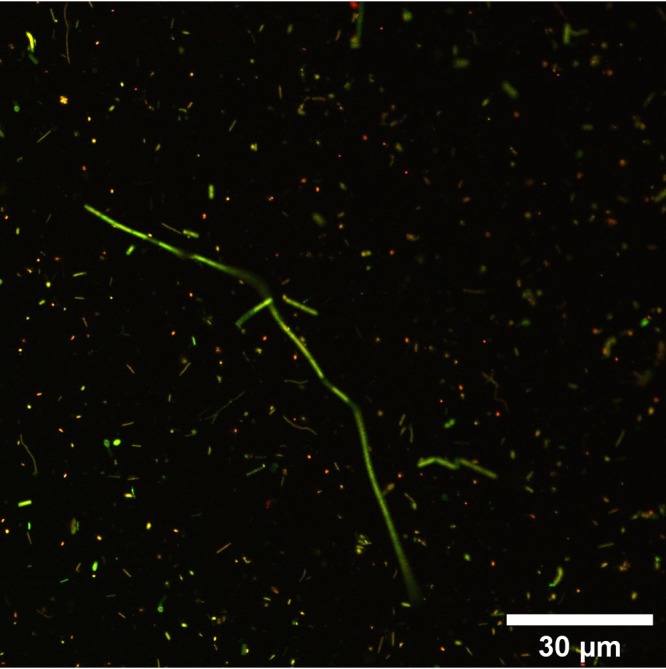
CLSM fluorescence micrograph of a water droplet isolated from the La Brea Tar Pits. The cells were stained with Syto 9 and propidium iodide. Membrane-intact cells appear green, whereas membrane-damaged cells are stained red.

**FIG 4:**
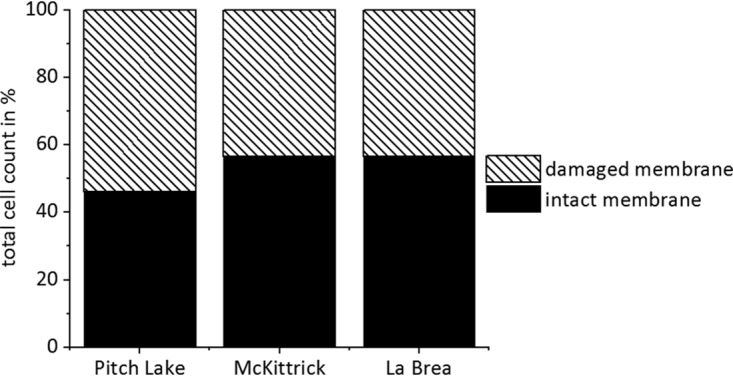
Distribution of membrane-intact and membrane-damaged cells in water droplets of the three tested oil seeps. In total, 197 cells were evaluated from Pitch Lake droplets, 1,394 from McKittrick droplets, and 1,564 from La Brea droplets.

**FIG 5:**
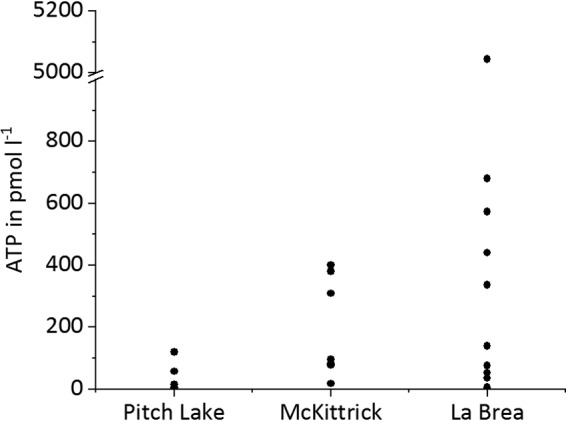
Measured ATP contents in water droplets extracted from the three oil seeps.

To analyze whether the observed cells were living microorganisms, we applied LIVE/DEAD staining to differentiate between membrane-intact and membrane-damaged cells (Fig. 3). Membrane-intact cells (Fig. 3, green fluorescent signal) were found in all populated droplets. The ratio between membrane-intact and membrane-damaged (Fig. 3, red fluorescent signal) cells varied between water droplets from the three oil seeps. Nevertheless, the average amount of intact cells was around 53% in all three seeps, indicating that substantial amounts of the observed cells were alive (Fig. 4). In dead controls, 98% of the cells were membrane damaged, indicating the reliability of the method (results not shown). Furthermore, we determined the concentrations of ATP, which is an indicator for active and live cells, in single droplets. Control ATP standards dissolved in water or water extracted from Pitch Lake oil did not indicate either inhibition or enhancement of the signal obtained (data not shown). ATP was detected in most of the tested droplets, but the average ATP concentration in extracted droplets varied within and between the three oil seeps (Fig. 5). The lowest average ATP concentration appeared in droplets from Pitch Lake, with 21.8 pM, followed by McKittrick, with 194.8 pM, and La Brea, with 492.2 pM.

### Microbial community analysis.

16S rRNA gene sequencing was used for evaluating similarities between the three oil seeps which might reveal a core community of typical oil-degrading microorganisms living in the water droplets. Bacterial and archaeal community compositions were analyzed by 16S rRNA gene sequencing of 10 to 12 separate water droplets from each oil seep. After read processing, quality filtering, and rarifying every sample to 14,281 reads, 4.6 million sequences were recovered across all samples. Sequences were clustered into 558 operational taxonomic units (OTUs) at a 97% sequence similarity cutoff. Among those, 525 OTUs belonged to 26 bacterial phyla and 33 OTUs to 4 archaeal phyla. The individual water droplets contained between 64 and 316 OTUs each.

Typical microbial inhabitants of oil reservoirs were found in all water droplets, indicating that the water droplets originated from the reservoir and were not introduced from the surface of the oil seep. The most prominent representatives belonged to the bacterial phyla *Proteobacteria*, *Bacteroidetes*, *Firmicutes*, *Synergistetes*, *Deferribacteres*, *Thermotogae*, *Chloroflexi*, *Bacteroidia*, and candidate phylum “Atribacteria,” while *Euryarchaeota* and *Woesearchaeota* represented the dominant archaeal phyla. OTUs that could not be classified by the RDP classifier were reclassified using BLAST against the nonredundant NCBI nucleotide database (17). Most unclassified OTUs could be assigned to the candidate phyla “Atribacteria” and “Parcubacteria.”

**FIG 6:**
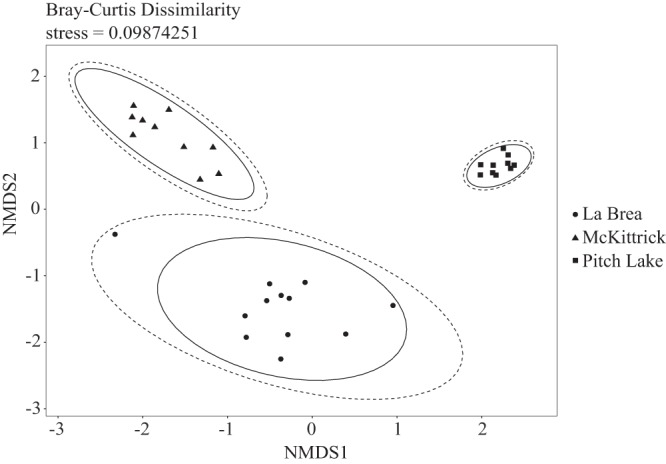
Nonmetric multidimensional scaling (NMDS) plot of beta diversity of all 32 water droplet communities from the three oil seeps. NMDS was calculated based on Bray-Curtis dissimilarity indices with stress level of 0.098. Dashed ellipses display the normal distribution, and solid ellipses display the *t* distribution.

The 10 most abundant OTUs in the respective oil seeps represented 8.38%, 4.45%, and 7.45% of Pitch Lake, McKittrick, and La Brea overall droplet communities, respectively, indicating that the communities were not dominated extensively by individual OTUs (Table 1). This is supported by the Simpson diversity indices of *D* = 0.75 ± 0.11 (mean ± standard deviation) for Pitch Lake, *D* = 0.94 ± 0.02 for McKittrick, and *D* = 0.80 ± 0.18 for La Brea droplets, which point at rather evenly distributed and, thus, relatively diverse communities. Alpha diversities by Shannon-Wiener indices of *H* = 2.2 ± 0.5 for Pitch Lake droplets, *H* = 3.7 ± 0.2 for McKittrick droplets, and *H* = 2.7 ± 1.0 for La Brea droplets indicate the most diverse community in McKittrick droplets. The compositional differences between the droplet communities were calculated as Bray-Curtis dissimilarities and indicate that the individual droplet community compositions were more similar within the respective oil seeps, leading to a clustering of the three seeps separately from each other (Fig. 6). Among the 558 OTUs identified in the three oil seeps investigated, 88 OTUs (16%) were found in all three oil seeps. Furthermore, 8 of these were present in 97 to 100% of the analyzed droplets, building a significant core community. This core community covered relative abundances of 3.18% (Pitch Lake), 1.22% (McKittrick), and 1.60% (La Brea) of the droplet communities within the respective oil seeps. Furthermore, La Brea and McKittrick shared 185 OTUs (33%), La Brea and Pitch Lake had 17 OTUs (3%) in common, and McKittrick and Pitch Lake had 11 OTUs (2%) in common. Even though many OTUs were present in all three oil seeps, the relative abundances of each OTU varied greatly between droplets within each oil seep. Nevertheless, the large percentage of the core community indicates a high degree of specialization. Pitch Lake droplets contained 31 unique OTUs, McKittrick 79 unique OTUs (14%), and La Brea 147 unique OTUs (26%). The top 10 OTUs of each individual droplet based on the family level are shown in Fig. S1 in the supplemental material.

**TABLE 1:** The 10 most abundant OTUs within each oil seep, together with their relative abundances in descending order

OTU	Family	Genus	% prevalence^*a*^	Mean relative abundance (±SD) of OTUs in^*b*^:		
				La Brea	McKittrick	Pitch Lake
1	*Hydrogenophilaceae*	*Tepidiphilus*		BD	0.01 (0.004)	3.8 (0.16)
2	*Porphyromonadaceae*	Unclassified	100	1.01 (0.08)	0.66 (0.04)	0.9 (0.06)
3	*Comamonadaceae*	Unclassified		1.47 (0.2)	0.66 (0.08)	BD
4	*Methanotrichaceae*	*Methanothrix*		1.8 (0.13)	BD	BD
5	*Pseudomonadaceae*	*Pseudomonas*		0.93 (0.24)	0.03 (0.01)	BD
6	*Clostridiales_Incertae_Sedis_XI*	*Soehngenia*	97	0.01 (0.001)	0.05 (0.003)	0.86 (0.06)
7	*Betaproteobacteria* (unclassified)	Unclassified		BD	0.7 (0.05)	BD
8	*Desulfobulbaceae*	*Desulfoprunum*		0.01 (0.002)	0.55 (0.05)	BD
9	*Syntrophobacteraceae*	Unclassified		BD	0.51 (0.08)	BD
10	Woesearchaeota (unclassified)	Unclassified		BD	BD	0.56 (0.06)
11	*Syntrophorhabdus*	Unclassified		0.38 (0.06)	BD	BD
12	*Comamonadaceae*	Unclassified		BD	0.01 (0.002)	0.53 (0.17)
13	*Hydrogenophilaceae*	*Tepidiphilus*		0.43 (0.04)	0.01 (0.002)	BD
14	*Hydrogenophilaceae*	*Thiobacillus*		BD	0.4 (0.03)	BD
15	*Deferribacteraceae*	Unclassified	97	0.02 (0.003)	0.08 (0.01)	0.55 (0.11)
16	*Synergistaceae*	*Anaerobaculum*		0.32 (0.03)	0.01 (0.003)	BD
17	Atribacteria (unclassified)	Unclassified	100	0.16 (0.02)	0.03 (0.004)	0.2 (0.01)
18	*Gammaproteobacteria* (unclassified)	Unclassified		0.45 (0.13)	BD	BD
19	*Synergistaceae*	*Thermovirga*		BD	BD	0.43 (0.02)
20	*Deferribacteraceae*	*Calditerrivibrio*		0.32 (0.05)	0.01 (0.001)	BD
22	Bacteria (unclassified)	Unclassified		0.33 (0.03)	0.01 (0.001)	BD
23	*Petrotogaceae*	Unclassified	97	0.01 (0.002)	0.003 (0.001)	0.39 (0.02)
24	*Syntrophaceae*	*Desulfomonile*		BD	0.29 (0.04)	BD
25	*Bacillaceae_1*	Unclassified	100	0.07 (0.01)	0.19 (0.02)	0.003 (0.0003)
26	*Porphyromonadaceae*	*Proteiniphilum*		BD	0.26 (0.02)	BD
27	*Bacteroidaceae*	*Bacteroides*		BD	0.23 (0.02)	BD
45	*Petrotogaceae*	Unclassified		BD	BD	0.15 (0.01)
21	*Synergistaceae*	*Anaerobaculum*	100	0.17 (0.03)	0.05 (0.01)	0.1 (0.01)
36	Bacteria (unclassified)	Unclassified	100	0.05 (0.01)	0.06 (0.01)	0.06 (0.003)
52	*Bacillaceae_1*	Unclassified	97	0.03 (0.004)	0.07 (0.01)	0.001 (0.0002)
38	*Anaerolineaceae*	Unclassified	97	0.06 (0.01)	0.02 (0.002)	0.1 (0.01)

a A core community of 10 OTUs was defined as present in 97 to 100% of the droplets of all three sites (prevalence). As part of the core community, OTUs 21, 36, 52, and 38 were added to the table regardless of their respective relative abundance.

b The 10 most abundant OTUs within each oil seep are marked in light gray. OTUs which were not detected in the particular seep or were detected with an abundance of <0.009% are marked as below detection (BD). Data depict the mean value and standard deviation of the relative abundances of the respective organism in all droplets from one site.

## DISCUSSION

The discovery of microorganisms in tiny water droplets suspended in oil of Pitch Lake in Trinidad, Trinidad and Tobago, revealed a new habitat for microorganisms and a new concept for oil degradation (15). However, it was still unclear if the finding of microorganisms in water droplets dispersed in oil was a unique observation from Pitch Lake in Trinidad or if life in oil is a generic feature of oil reservoirs. Therefore, we sampled three natural oil seeps located at Pitch Lake in Trinidad, the McKittrick oil field in California, and the La Brea Tar Pits in Los Angeles, CA, to look for such water droplets. Furthermore, we aimed at characterizing the principal structures of the microbial communities in this extreme environment.

Indeed, similar small water droplets of 1 to 10 μl in size were found in all three natural oil seeps. Cell counting of the suspended microorganisms in the droplets indicated dense populations up to 1.2 × 10^6^ cells μl^−1^. This is an astonishing density compared to other deep subsurface habitats that only contain, for example, a thousand-fold fewer cells with around 10^5^ to 10^6^ cells cm^−3^ (corresponding to 10^2^ to 10^3^ cells μl^−1^), depending on the depth (1). With densities of 1 × 10^4^ to 4.25 × 10^4^ cells ml^−1^, the microbial abundance in production water from oil reservoirs is also much lower than in our droplets (9, 18). Moreover, the micrographs of our droplets indicated that some microorganisms seemed to grow at the oil-water interface of the droplets. Biofilm formation on hydrocarbon-oil interfaces was shown earlier for microbial degradation of alkanes or polycyclic aromatic hydrocarbons (19, 20). Hence, it is likely that the microorganisms in the water droplets form biofilms at the oil-water interface, which possibly increases the bioavailability and facilitates the degradation of *n*-alkanes (C_8_ to C_28_) and *n*-alcohols (C_12_ and C_16_) by sorption of hydrocarbons to extrapolymeric substances.

The microorganisms in water droplets of all three oil seeps were not only present in high densities but also alive, as indicated by a large portion of membrane-intact cells. Due to the technical limitations of the LIVE/DEAD assay, the true number of intact cells was most likely higher than the estimated 60%. Furthermore, metabolic activity could be shown by the presence of ATP, which is a constant value for living cells because microorganisms have to sustain an energy homoeostasis (21). With approximately 1.47 × 10^−21 ^mol ATP per cell, the microorganisms in the water droplets contained small quantities of ATP, indicating very little activity compared to the levels in other environmental habitats, which ranged from 10^−21^ to 10^−15 ^mol ATP per cell (21–23). Since ATP is rapidly consumed in the presence of biomass, we conclude that the ATP detected stemmed from living cells (21, 23–25). Hence, the results of LIVE/DEAD staining and the ATP determination indicate that the microorganisms detected in the water droplet were alive and active and not only dead microbes that were accidentally entrapped in the droplets.

The microbial community compositions showed similarities in the three oil seeps tested, and the calculated low Shannon-Wiener and high Simpson diversity indices are similar to those of other oil field microbial communities (26–29). Such values may reflect high specialization and long isolation of the communities, leading to reduced diversity but evenly composed microbial communities in the three sampled oil seeps. However, the low Shannon diversity is also certainly a consequence of the small sample size. It is anyway problematic to compare diversities of samples from different studies if they have not been rarified or normalized to a common size of the data set.

Although the communities in the droplets were clearly more similar within one seep than compared to the other two seeps, they shared a significant number of OTUs despite the fact that they are located hundreds (La Brea and McKittrick) or thousands (La Brea, McKittrick, and Pitch Lake) of kilometers away from each other. Most of these core OTUs were shared between the La Brea and McKittrick oil seeps (33%), which are geographically closer to each other, but La Brea or McKittrick also shared 16% of all detected OTUs with the Pitch Lake droplets. These commonalities between the three different seeps support the paradigm of Baas-Becking, “everything is everywhere, but the environment selects” (30, 31), especially since oil reservoirs represent a highly selective and extreme environment. Although, in principle, core communities can also consist of microorganisms that are not essential to the habitat, e.g., when samples are exposed to strong microbial dispersal (32, 33), this possibility is unlikely for the water droplets because they constitute highly isolated ecosystems that have probably been separated from each other over longer time scales (15).

These conclusions are supported by comparing the droplet communities to microbiomes found in other oil fields. The most abundant families from our droplets occurred in all three reservoirs and contained anaerobic or facultatively anaerobic members, which were also reported for other oil reservoirs all over the world at mesophilic to thermophilic conditions (5–7, 34–56).

The finding that water droplets populated with active microbial communities are found in the three oil seeps tested is a strong indication that life in water droplets dispersed in oil could be a generic feature of oil reservoirs. Moreover, the remarkable similarities of the microbial communities in physically isolated water droplets of geographically very distant oil seeps indicate that this microbial life is highly adapted.

## MATERIALS AND METHODS

### Oil sampling.

Natural asphalt and heavy oil (14, 57–59) were sampled from Pitch Lake (10°14′0.6882N″, 61°37′44.5638″W) on the island of Trinidad in Trinidad and Tobago, the La Brea Tar Pits (34°03′49.7″N, 118°21′25.1″W) in Los Angeles, CA, USA, and an unnamed oil seep (35°17′35.2″N, 119°38′10.5″W) on the McKittrick oil field, CA, USA. The distances are 180 km between La Brea and McKittrick, 6,322 km between La Brea and Pitch Lake, and 6,462 km between McKittrick and Pitch Lake. Oil surface temperatures during sampling were 36°C at Pitch Lake, 20.5°C at La Brea, and 20.5°C at McKittrick. All spots are natural oil seeps where heavily degraded oil reaches the surface.

Oil was sampled from different spots on each particular oil seep (6 spots at Pitch Lake, 1 spot at McKittrick, and 3 spots at La Brea). Samples were taken with 50-ml syringes, where the tip of the syringe was cut off with a scalpel, and transferred into separate sterile glass jars (63 samples at Pitch Lake, 30 samples at McKittrick, and 12 samples at La Brea), flushed on site directly after sampling with N_2_ (5.0 grade; obtained from Massy Gas Products, Savonetta Estate, Trinidad and Tobago, for Pitch Lake and from Tyms, Inc., Los Angeles, CA, USA, for La Brea and McKittrick), and closed with a sterile gastight-sealed lid. The jars were shipped to the laboratory by airfreight and stored at 4°C until further use.

### Droplet *in situ* observations.

For visualization of cells in water droplets, the oil containing the microhabitats was transferred to hanging-drop slides (Brand, Wertheim, Germany) using spatulas. The cavities of the slides were used as reservoirs to avoid compression of the oil during microscopy. Cells were stained with 2 μl of a Syto 9 solution (10 μM; Molecular Probes, Eugene, OR, USA) by pipetting directly into visible water droplets, thereby increasing the original droplet volume. After injection, the samples were covered with a cover slide and incubated in the dark for 20 min. A confocal laser scanning microscope (TCS SP8 HCS A; Leica Microsystems) equipped with a 488-nm argon laser and an HC PL APO 63×/1.4 numeric aperture (NA) CS2 oil objective was used for visualizing the cells. Images of the Syto 9-stained cells were taken with an excitation wavelength of 488 nm and an emission range from 507 to 550 nm. LAS.X (version 3.5.2) and ImageJ (version 1.52i) software with the Bio-Formats plug-in (version 5.8.2) were used for data processing.

### Droplet sampling.

For droplet extraction, oil samples were heated for ∼30 min at 45°C to render the oil more liquid and to allow the lighter water droplets to ascend to the sample surface. Since the average oil temperature of the sampling spots was about 31°C, and in some cases up to 43.9°C, cell damage due to heating was deemed unlikely. Subsequently, oil samples were cooled to room temperature and water droplets were collected from the sample surface with 10-μl pipettes.

### Cell counting in individual droplets.

For cell counting, 1 μl of each water droplet was diluted in 39 μl of water (18.2 MΩ · cm water resistivity using a Milli-Q Advantage A10 device equipped with a Q-GardT2 filter, a QuantumTEX filter, and a MillipakExpress 40 0.22-μm filter; Merck Millipore, Germany). Cells were counted with a light microscope (DMLS; Leica, Germany) equipped with a 40×/0.65 NA ocular (C Plan; Leica, Germany) and with a counting chamber (Thoma; Brand GmbH + Co. KG, Germany). In total, 10 droplets from each oil seep were examined.

To validate the first counting, an additional 12 droplets from Pitch Lake were stained with 4′,6-diamidino-2-phenylindole (DAPI). To this end, 1 μl of each water droplet sampled from Pitch Lake oil was mixed with 1 ml of DAPI solution (25 μg ml^−1^; Sigma, Steinheim, Germany), incubated for 20 min in the dark, and subsequently filtered through 0.2-μm polycarbonate membrane filters (Isopore; EMD Millipore, Cork, Ireland). Filters were stored at 4°C until further use. Cells were counted with an epifluorescence microscope (Axio scope.A1; Carl Zeiss Microscopy GmbH, Göttingen, Germany) equipped with a 100×/1.25 NA oil objective (N-Achroplan; Carl Zeiss Microscopy GmbH, Göttingen, Germany).

### Determination of cell membrane integrity in individual droplets.

The membrane integrity of cells isolated from water droplets was investigated with the LIVE/DEAD BacLight bacterial viability kit (Molecular Probes, Eugene, OR, USA). The membrane permeability of propidium iodide can be increased by too high propidium iodide concentrations or other influences, such as oxygen, heat, or cells being in their division cycle, leading to an overestimation of membrane-damaged cells (60–63). To avoid false-negative staining results due to overstaining with propidium iodide, different propidium iodide concentrations were tested by staining an unpublished sulfate-reducing enrichment culture from Pitch Lake (Table S1). The manufacturer’s instructions were modified according to the test results (not shown), and the staining reagent concentrations were adjusted to 1.65 mM Syto 9 and 0.05 mM propidium iodide, respectively.

Isolated droplets from Pitch Lake, McKittrick, and La Brea Tar Pits were diluted in 1 ml of substrate-free freshwater medium (64) (for Pitch Lake) or phosphate-buffered saline (pH 7.5) (for McKittrick and La Brea). Then, 3 μl of staining reagent was added to each droplet, followed by incubation for 20 min at room temperature in the dark. For dead controls, approximately 15 μl droplet water was pooled and two 2-μl amounts of the mixture were each diluted in 1 ml 70% isopropanol (BioReagent for molecular biology; Sigma-Aldrich, St. Louis, MO, USA). The controls were incubated for 1 to 2 h at 60°C and 900 rpm in a thermoshaker (ThermoMixer X; Eppendorf AG, Hamburg, Germany). Afterwards, all samples were filtered through 0.2-μm polycarbonate membrane filters (Isopore; EMD Millipore, Cork, Ireland). The filters were stored at 4°C in the dark. Two confocal laser scanning microscopes were used for visualizing microorganisms in the water droplets. The Axiovert 100 M microscope (Carl Zeiss Microscopy GmbH, Göttingen, Germany) was equipped with a 100×/1.3 NA Plan-NeoFluar oil objective, LP 385 and LP 650 filters, a 488-nm argon laser, and LSM 510 software; the TCS SP8 HCS A microscope (Leica Microsystems, Germany) was equipped with an HC PL APO 63×/1.4 NA CS2 oil objective, a 488-nm and 514-nm argon laser, and LAS.X software (version 3.5.2). Images of the Syto 9-stained cells were taken with an excitation wavelength of 488 nm and an emission range from 507 to 550 nm. Images of propidium iodide were taken with an excitation wavelength of 514 nm and an emission range from 617 to 680 nm. ImageJ (version 1.52i) software with the Bio-Formats plug-in (version 5.8.2) was used for analysis.

### ATP quantification.

ATP (ATP) in the isolated water droplets was quantified with the BacTiter-Glo microbial cell viability assay (Promega, Madison, WI, USA) according to the manufacturer’s instructions. From each isolated droplet, 3 μl was diluted in 97 μl water and mixed with 100 μl BacTiter-Glo reagent. After 5 min of incubation, all samples were measured with a luminometer (Glomax 20/20 luminometer; Promega, Sunnyvale, CA, USA).

To exclude possible matrix effects during measurements of hydrocarbon-rich water, inhibition tests were performed with matrix water. To this end, oil was heated to 80°C, transferred into 50-ml centrifuge tubes, and subsequently centrifuged for 2 h at 3,214 × *g* (5810 R centrifuge; Eppendorf, Hamburg, Germany). After centrifugation, approximately 200 μl of water could be extracted from the approximately 60 ml of oil and mixed with 200 μl of BacTiter-Glo reagent (Promega, Madison, WI, USA). The solution was incubated overnight for full ATP removal. For luciferase inactivation, the mixture was heated twice for 10 min in a thermoshaker (ThermoMixer X; Eppendorf AG, Hamburg, Germany) at 95°C and 900 rpm. Afterwards, the ATP- and luciferase-free matrix water was diluted with water (18.2 MΩ · cm, 3% final concentration [vol/vol], equivalent to sample volume). The processed matrix water served as the solvent for 10 mM ATP (Promega, Madison, USA), used as a reference standard.

### DNA extraction, 16S rRNA gene amplification, library preparation, and sequencing.

We developed a protocol consisting of two lysis steps for the extraction of DNA from tiny water droplets with a volume as small as 1 μl. In order to lyse Gram-positive bacteria, 1 μl of an enzyme cocktail was mixed with 1 μl of droplet water and incubated for 1 h at 37°C. The enzyme cocktail consisted of 2.5 U μl^−1^ lysozyme (Sigma-Aldrich, USA), 0.6 U μl^−1^ mutanolysin (Sigma-Aldrich, USA), and 0.048 U μl^−1^ lysostaphin (Sigma-Aldrich, USA) and was designed to achieve an unbiased representation of the microbial community based on the data published in reference 65. In order to lyse Gram-negative bacteria and archaea, 2 μl of alkaline solution was added and the mixture was incubated for 5 min at room temperature. The alkaline solution contained 0.4 M KOH (VWR, Darmstadt, Germany) and 0.1 M dithiothreitol (Sigma-Aldrich, USA) (66). Alkaline lysis was stopped by adding 2 μl Tris-HCl (pH 4) (Fisher Scientific, Schwerte, Germany).

Amplification of the 16S rRNA genes, library preparation, and sequencing were performed on two technical replicates per DNA sample. The 16S rRNA gene library preparation was accomplished according to the Illumina 16S *Metagenomic Sequencing Library Preparation* guide (part number 15044223 rev. B) with the following modifications. 16S rRNA gene sequences were amplified by targeting the hypervariable V3–V4 region with forward primer Pro341f (5′-CCT ACG GGN BGC ASC A-3′) and an overhang adaptor (5′-TCG TCG GCA GCG TCA GAT GTG TAT AAG AGA CAG CCT ACG GGN BGC ASC A-3′) and with reverse primer Pro805r (5′-GAC TAC NVG GGT ATC TAA TCC-3′) and an overhang adaptor (5′-GTC TCG TGG GCT CGG AGA TGT GTA TAA GAG ACA GGA CTA CNV GGG TAT CTA ATC C-3′). The choice of primers and hypervariable region aimed at covering the broadest possible spectrum of both bacteria and archaea (67, 68). Amplicon PCRs were performed in reaction mixture volumes of 25 μl, each containing 2 μl of extracted DNA, 12.5 μl of 2× KAPA HiFi hot start ready mix (KAPA Biosystems, MA, USA), and 0.25 μM each primer with overhang adaptor. The thermocycling protocol started with 5 min at 95°C, followed by a touchdown protocol with 10 cycles of 30 s at 95°C, 30 s at 60 to 55°C, with a decline of 0.5°C per cycle, and 30 s at 72°C, continuing with 30 cycles of 95°C for 30 s, 54°C for 30 s, and 72°C for 30 s, and then a final extension at 72°C for 10 min. Amplicon PCR products were checked by agarose gel electrophoresis with 1% (wt/vol) gels. Purification of amplicons proceeded according to the Illumina protocol using 16 μl of MagSi-NGS Prep-Plus magnetic beads for 20 μl of PCR product (Steinbrenner Laborsysteme GmbH, Mannheim, Germany). Purified samples were employed as templates for index PCRs using the Nextera XT index kit version 2 set D (FC-131-2004; Illumina, USA) and the following thermocycling protocol: 95°C for 3 min, then 10 cycles with 95°C for 30 s, 55°C for 45 s, and 72°C for 60 s, and a final extension at 72°C for 5 min. Index PCR products were checked by agarose gel electrophoresis and purified with magnetic beads as described above. The DNA concentration of each sample was quantified using the Qubit double-stranded DNA (dsDNA) high-sensitivity (HS) assay kit (Invitrogen, USA) and normalized to 4 ng μl^−1^ using 10 mM Tris-Cl, pH 8.5 (buffer EB; Qiagen, Germany). About 96 normalized samples were combined in one tube and submitted to the sequencing company (Eurofins Genomics Germany GmbH, Germany) for sequencing on the Illumina MiSeq platform. Sequencing reads were demultiplexed by the sequencing facility.

Bioinformatic analysis was carried out using mothur (version 1.40.5, last updated 19 June 2018) MiSeq standard operating procedure (SOP) (69, 70). After merging forward and reverse reads, sequences with ambiguous bases, shorter than 380 bp or longer than 470 bp, were removed from the data set. All remaining unique sequences were aligned to the bacterial database SILVA, version 132, customized to the region of interest (71–73). Chimeras and nonribosomal sequences were removed and taxonomic classification was assigned based on RDP, trainset 16 (Ribosomal Database Project) (74). Sequences were clustered into operational taxonomic units (OTUs) by defining a 97% similarity cutoff (setting of 0.03 distance limit). Reads were rarified via mothur to the lowest detected read number of 14,281 of sample 46_PL (Pitch Lake). The R package phyloseq (75) was applied for diversity and community analysis of rarefied samples. OTUs with a read number below 10 and OTUs which were only abundant in one of the two technical replicates were rated as rare species or sequencing mistakes and removed from the data set. Afterwards, technical replicates were pooled by calculating the mean number of reads for each OTU.

### Data availability.

Raw sequencing reads were deposited in the NCBI database in BioProject under accession number PRJNA546121.

## Supplementary Material

Supplemental file 1
