# Supplementary material for: Densely Populated Water Droplets in Heavy-Oil Seeps
Source: Appl Environ Microbiol. 2020 May 19;86(11):e00164-20. doi: 10.1128/AEM.00164-20 (PMC7237766; doi:10.1128/AEM.00164-20)
Supplement: Supplemental file 1 [file AEM.00164-20-s0001.pdf]

Appendix

Community composition per droplet

Typical microbial inhabitants of oil reservoirs were found in all water droplets.

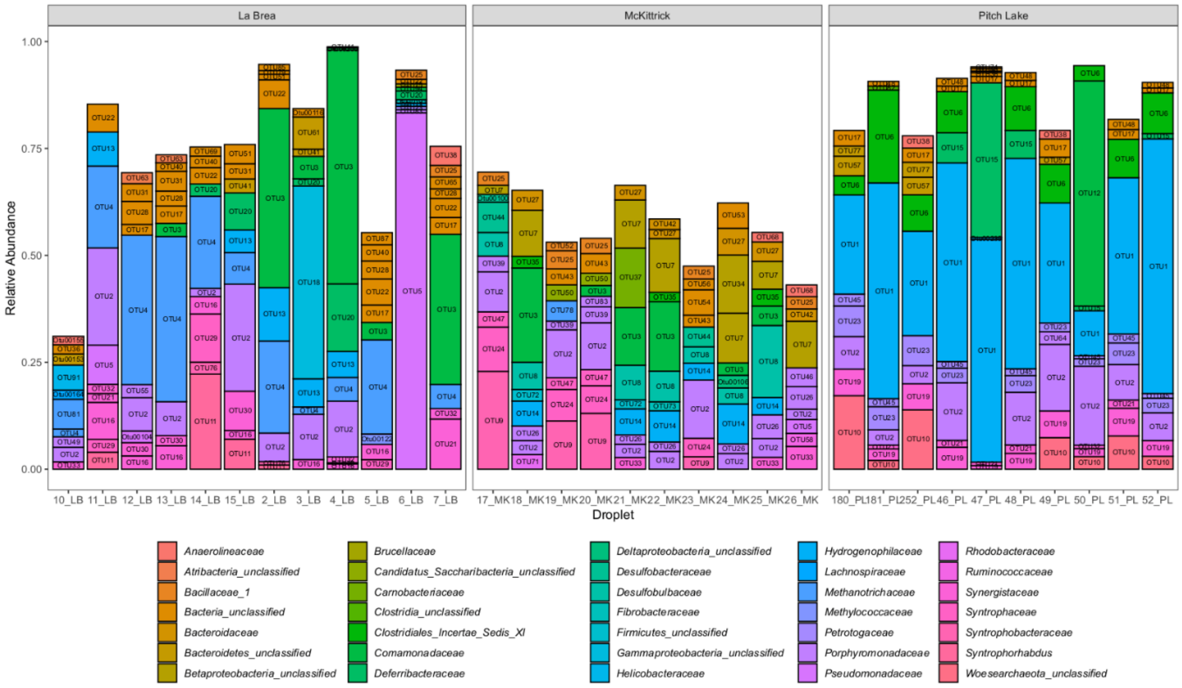

Fig. S1. The 10 most abundant OTUs per individual droplet community. Boxes represent OTUs colored by family.

LIVE/DEAD-Staining method validation

To avoid false negative staining results due to overstaining of propidium iodide, the manufacturer's instructions were modified according to Frösler et al., 2017 (1). Different propidium iodide concentrations (shown in table S1) were tested and a sulfate-reducing, phenanthrene-degrading mixed culture isolated from the Pitch Lake served as positive control. A culture treated with 70 % isopropanol (99.5 % for molecular biology; Sigma-Aldrich, St. Louis, USA) for 1 h, served as dead control.

Table S1 shows the tested propidium iodide concentrations for method validation

Syto®9/PI mixed in ratio 1:1				
	Test 1	Test 2	Test 3	Test 4
Syto®9	3.34 mM	3.34 mM	3.34 mM	3.34 mM
PI	18.3 mM	0.366 mM	0.183 mM	0.092 mM

References

1. Frösler J, Panitz C, Wingender J, Flemming H-C, Rettberg P. 2017. Survival of Deinococcus geothermalis in biofilms under desiccation and simulated space and martian conditions. Astrobiology 17:431-447.
